# A case of acute acalculous cholecystitis after pulmonary vein isolation—novel phenotype of perioesophageal vagal nerve injury: a case report

**DOI:** 10.1093/ehjcr/ytaf204

**Published:** 2025-04-29

**Authors:** Satoshi Ishii, Junya Hosoda, Kohei Iguchi, Kazuki Fukui, Kiyoshi Hibi

**Affiliations:** Department of Cardiology, Yokohama City University Hospital, 3-9 Fukuura, Kanazawa-ku, Yokohama 236-0004, Japan; Department of Cardiology, Yokohama City University Hospital, 3-9 Fukuura, Kanazawa-ku, Yokohama 236-0004, Japan; Department of Cardiology, Kanagawa Cardiovascular and Respiratory Center, 6-16-1 Tomioka-higashi, Kanazawa-ku, Yokohama 236-0051, Japan; Department of Cardiology, Kanagawa Cardiovascular and Respiratory Center, 6-16-1 Tomioka-higashi, Kanazawa-ku, Yokohama 236-0051, Japan; Department of Cardiology, Yokohama City University Hospital, 3-9 Fukuura, Kanazawa-ku, Yokohama 236-0004, Japan

**Keywords:** Acute acalculous cholecystitis, Pulmonary vein isolation, Perioesophageal vagal nerve injury, Case report

## Abstract

**Background:**

Perioesophageal vagal nerve injury related to pulmonary vein isolation using radiofrequency catheter ablation sometimes causes somatic symptoms including gastric dilation and motility disorder. However, reports of acute acalculous cholecystitis after pulmonary vein isolation are rare.

**Case summary:**

We report a case of a 64-year-old man diagnosed with paroxysmal atrial fibrillation. No acute complications occurred on the day of the procedure, but he complained of epigastric pain 27 h after the ablation procedure. He was diagnosed with mild acute acalculous cholecystitis and underwent laparoscopic cholecystectomy.

**Discussion:**

Vagal nerve injury appears to be related to the development of acute acalculous cholecystitis because many patients with acute cholecystitis after abdominal surgery along with resection of vagal nerve have acute acalculous cholecystitis. We speculate that acute acalculous cholecystitis after pulmonary vein isolation is one phenotype of perioesophageal vagal nerve injury.

**Learning points**

- Among several complications related to pulmonary vein isolation, perioesophageal vagal nerve injury sometimes causes somatic symptoms including gastric dilation and motility disorder.
- Our case suggests that it is necessary to recognize that acute acalculous cholecystitis is one phenotype of perioesophageal vagal nerve injury related to pulmonary vein isolation with radio frequency catheter ablation.

## Introduction

Pulmonary vein isolation using radiofrequency catheter ablation is an effective and established treatment strategy in patients with atrial fibrillation. Among several complications related to pulmonary vein isolation,^1^ perioesophageal vagal nerve injury sometimes causes somatic symptoms including gastric dilation and motility disorder.^2^ On the other hand, acute acalculous cholecystitis occurs in 10% of cases of acute cholecystitis, which is caused by cholestasis in the gallbladder, gallbladder ischaemia after abdominal surgery, multiple trauma, burn, and long-term total parenteral nutrition.^3^ In particular, many patients with acute cholecystitis after abdominal surgery along with resection of vagal nerve have acute acalculous cholecystitis. Therefore, vagal nerve injury may be related to the development of acute acalculous cholecystitis.^4^ We report a case of acute acalculous cholecystitis that presented on the day following pulmonary vein isolation with radiofrequency catheter ablation in a patient with paroxysmal atrial fibrillation.

## Summary figure

**Table:** 

Time	Events
4 months prior	Presented to our hospital with paroxysmal atrial fibrillation.
Day0	
9:00	The patient was transferred to the angiography room
9:45	Procedure initiated
10:20	Radiofrequency catheter ablation started
11:15	Ablation completed and intravenous anaesthesia stopped
Day1	
9:00	Blood tests the next morning showed no abnormal findings.
14:00	The patient complained of epigastric pain. Enhanced computed tomography showed gallbladder wall thickening, subserosal edema, and gallbladder enlargement without any stones inside. His serum CRP level was 2.19 mg/dL. He was diagnosed with mild acute acalculous cholecystitis and underwent laparoscopic cholecystectomy.
Day5	He Was Discharged. Pathological Examination Revealed Acute Acalculous Cholecystitis.

## Case report

A 64-year-old man who had been diagnosed with paroxysmal atrial fibrillation (CHA₂DS₂-VASc Score 1 point, and HAS-BLED Score 1 point) 4 months ago entered our hospital to undergo radiofrequency catheter ablation. He had a history of hypertension, hyperuricaemia, and obstructive sleep aponea syndrome, which was treated with continuous positive airway pressure. He did not have a history of abdominal surgery. Apixaban was administered without antiarrhythmic drugs or proton pump inhibitors. Transthoracic echocardiography showed left ventricular ejection fraction (LVEF) of 61%, left ventricular internal dimension (LVID) diastolic/systolic of 41/26 mm, left atrial dimension (LAD) of 41 mm, left atrial volume index (LAVI) of 38.8 mL/m^2^, and no valvular disease. By enhanced CT examination, left atrial volume was 205 mL, and the oesophagus was located just behind the left pulmonary vein.

**Figure 1:**
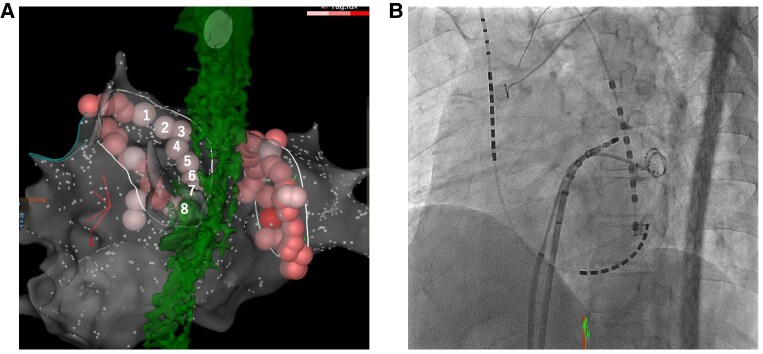
(*A*) Eight ablation lesions at the posterior aspect of the left pulmonary vein and the oesophagus is shown. (*B*) The positional relationship between the oesophageal temperature probe and the ablation catheter during ablation of the posterior aspect of the left pulmonary vein is shown.

**Figure 2:**
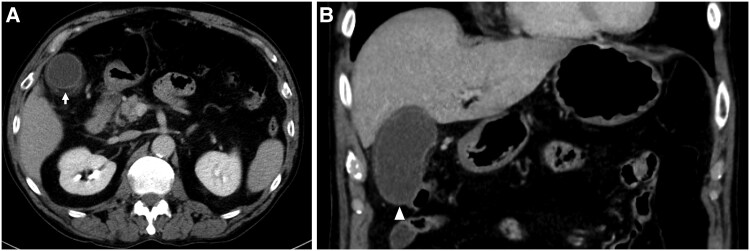
(*A*) Gallbladder wall thickening and subserosal oedema are shown (arrow). (*B*) Gallbladder enlargement without a gallbladder stone is shown (arrowhead).

The radiofrequency catheter ablation was performed using THERMOCOOL SMARTTOUCH™ Catheter (Biosense Webster Inc.) on three-dimensional electroanatomical mapping system (CARTO3, Biosense Webster Inc). The ablation procedure involved point-by-point pulmonary vein isolation and ablation index (AI) guided ablation aimed at 370 (30W) on the posterior aspect of the left pulmonary vein and 400 (35W) on the other aspects of the pulmonary veins, respectively. A multi-sensor oesophageal temperature probe was used for oesophageal temperature monitoring. The positional relationship between the oesophagus and eight ablation lesions (average contact force, 7–13 g; and time, 15.7–26.8 s) at the posterior aspect of the left pulmonary in CARTO and fluoroscopy is shown *Figure 1*. The oesophageal temperature did not rise up to 41°C. We finished the procedure, and no acute complications occurred on the day of the procedure. Blood test, electrocardiogram, chest radiography, and echocardiography performed the next morning showed no abnormal findings. However, the patient complained of epigastric pain 27 h after the ablation procedure. The abdomen was flat and soft, but tenderness was noted in the epigastric region, and Murphy's sign was positive. Enhanced computed tomography showed gallbladder wall thickening, subserosal oedema, and gallbladder enlargement without any stones inside (*Figure 2*). Gastric dilatation was not observed. His serum CRP level was 2.19 mg/dL (normal value 0–0.14) by peripheral blood examination. He was diagnosed with mild acute acalculous cholecystitis and underwent laparoscopic cholecystectomy. The postoperative course was uneventful, and he was discharged on the fourth day after surgery (on the fifth day after the ablation procedure). During the two-year follow-up period, no abdominal symptoms have recurred after cholecystectomy. Pathological examination revealed neutrophil infiltration in the mucous membrane without congestion, oedema, or necrosis. As expected, no stone was found in the gallbladder. Pathological examination revealed acute acalculous cholecystitis.

## Discussion

Perioesophageal vagal nerve injury is known as one of the complications of pulmonary vein isolation by radiofrequency catheter ablation in patients with atrial fibrillation. Many cases of perioesophageal vagal nerve injury present with acute gastric dilatation, which is caused by gastric hypomotility, convulsions of the pylorus of the stomach, and so on.^2^ On the other hand, for the first time, Tsuboi *et al*. reported two cases of acute acalculous cholecystitis after radiofrequency catheter ablation including pulmonary vein isolation, left atrium posterior wall isolation, and mitral isthmus linear ablation and implied that acute acalculous cholecystitis was caused by perioesophageal vagal nerve injury.^5^ Although Tsuboi *et al*. did not describe the details of ablation lesions on top of the oesophagus, we speculate the number of ablation lesions in the two cases was more than in our case because posterior wall isolation and mitral isthmus linear ablation were performed in the two cases.

**Figure 3:**
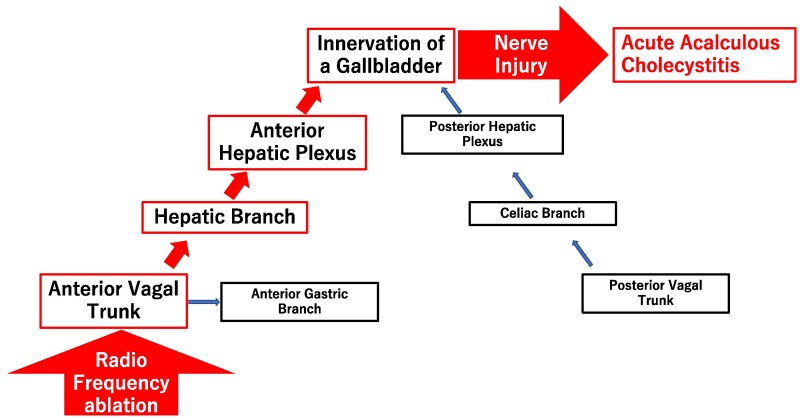
Relationship between radiofrequency ablation and acute acalculous cholecystitis.

Many patients with acute cholecystitis after abdominal surgery along with resection of the vagal nerve had acute acalculous cholecystitis. Therefore, vagal nerve injury appears to be related to the development of acute acalculous cholecystitis.^4^ The innervation of the gallbladder is predominantly composed of two routes of the anterior hepatic plexus and the posterior hepatic plexus.^6^ The anterior hepatic plexus contains the coeliac plexus and the branches arising from the hepatic division of the anterior vagal trunk located in front of the oesophagus. The posterior hepatic plexus contains the coeliac plexus and the coeliac branches arising from the posterior vagal trunk located behind the oesophagus. The perioesophageal vagal nerve close to the left atrium is the anterior vagal trunk. In this case, the ablation line of the posterior aspect of the left pulmonary vein was just on top of the oesophagus on enhanced CT. Therefore, we speculate that injury of the anterior vagal trunk by the ablation may have caused acute acalculous cholecystitis (*Figure 3*). Perioesophageal vagal nerve injury is commonly known as one of the complications of pulmonary vein isolation, and it should be recognized that acute acalculous cholecystitis is one phenotype of perioesophageal vagal nerve injury after pulmonary vein isolation. Because acute acalculous cholecystitis sometimes gets worse over time,^7^ early diagnosis is important. In this case, cholecystectomy was performed due to the patient’s normal general condition.

Yoshimura *et al*. reported that the mean contact force of an ablation lesion on just top of the oesophagus was significantly higher in patients with perioesophageal vagal nerve injury than in those without.^8^ In this case, the mean contact force of four ablation lesions on just top of the oesophagus was 12.0 g, but the maximum contact force of them were 12, 31, 52, and 60 g. It was possible that a high contact force was related to perioesophageal vagal nerve injury in this case.

In this case, perioesophageal vagal nerve injury occurred, although the oesophageal temperature did not rise up to 41°C. It remains unclear whether oesophageal temperature monitoring can reduce the incidence of perioesophageal vagal nerve injury.^9,10^ When an oesophageal temperature probe does not be accurately located close to ablation lesions, it may not be able to monitor the accurate temperature of the oesophagus just close to ablation lesions, and the actual oesophageal temperature may be higher than the monitored temperature. Therefore, when it is attempted to ablate the left atrium on just top of the oesophagus confirmed with CT or esophagography, in order to prevent perioesophageal vagal nerve injury, it might be needed to perform minimum ablation of the left atrium just on the top of oesophagus even if the monitored esophageal temperature does not rise. In recent years, it was reported that the use of an esophageal deviating device significantly reduced esophageal injury in radiofrequency catheter ablation.^11^ It is expected that their use will reduce perioesophageal vagal nerve injury.

## Conclusion

We reported a rare case of atrial fibrillation in which acute acalculous cholecystitis occurred after pulmonary vein isolation using radiofrequency catheter ablation. Our case suggests that it is necessary to recognize that acute acalculous cholecystitis is the one phenotype of perioesophageal vagal nerve injury related to pulmonary vein isolation with radiofrequency catheter ablation. When attempts are made to ablate the left atrium on just top of the oesophagus, it might be necessary to perform minimum ablation of the left atrium just on the top of the oesophagus and avoid excessive contact force even if an oesophageal temperature probe is used. The use of an oesophageal deviating device may help prevent perioesophageal vagal nerve injury.

## Lead author biography


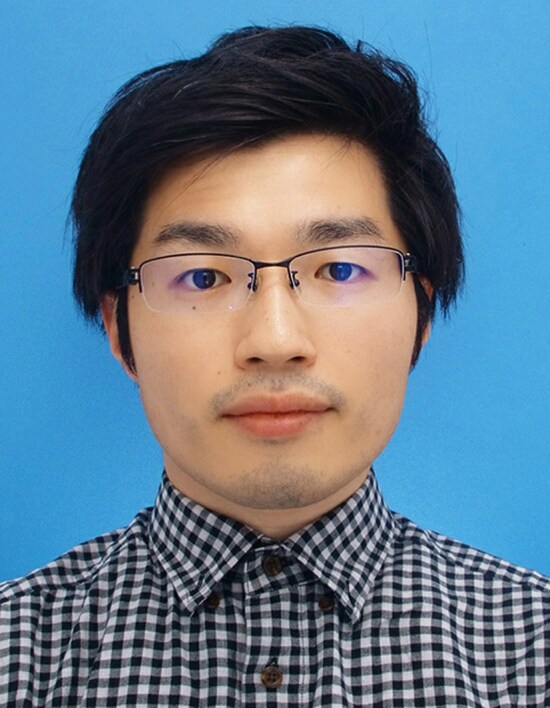


I graduated from Yokohama City University School of Medicine in 2016. I was employed in some hospitals as a cardiologist. At present, I am employed in the Yokohama City University Hospital Department of Cardiology as an electrophysiologist.

## Data Availability

The datasets generated and/or analysed during the current study are available from the corresponding author upon reasonable request.

## Notes

**Consent**: Written informed consent was obtained from the patient in accordance with COPE guidelines.

**Funding**: We had no funding for this case report.
